# Invasive Bacterial Infection in Patients with Interleukin-1 Receptor-associated Kinase 4 Deficiency: Case Report

**DOI:** 10.1097/MD.0000000000002437

**Published:** 2016-01-29

**Authors:** Hidetoshi Takada, Masataka Ishimura, Tomohito Takimoto, Toaki Kohagura, Hideto Yoshikawa, Masue Imaizumi, Koichi Shichijyou, Yoko Shimabukuro, Tomoo Kise, Nobuyuki Hyakuna, Osamu Ohara, Shigeaki Nonoyama, Toshiro Hara

**Affiliations:** From the Department of Perinatal and Pediatric Medicine, Graduate School of Medical Sciences, Kyushu University, Fukuoka, Japan (HT); Department of Pediatrics, Graduate School of Medical Sciences, Kyushu University, Fukuoka, Japan (MI, TT, TH); Department of Pediatrics, Naha City Hospital, Naha, Japan (TK); Department of Pediatrics, Nagaoka Rehabilitation and Medical Center for Children, Nagaoka, Japan (HY); Department of Hematology and Oncology, Miyagi Children's Hospital, Sendai, Japan (MI); Department of Pediatrics, Tokushima Red Cross Hospital, Komatsushima, Japan (KS); Okinawa Prefectural Nanbu Medical Center and Children's Medical Center, Shimajiri-gun, Japan (YS, TK); Center of Bone Marrow Transplantation, Hospital of University of the Ryukyu, Nakagami-gun, Japan (NH); Department of Technology Development, Kazusa DNA Research Institute, Kisarazu, Japan (OO); and Department of Pediatrics, National Defense Medical College, Tokorozawa, Japan (SN).

## Abstract

Interleukin-1 receptor-associated kinase 4 (IRAK4) deficiency (OMIM #607676) is a rare primary immunodeficiency of innate immune defect. We identified 10 patients from 6 families with IRAK4 deficiency in Japan, and analyzed the clinical characteristics of this disease.

Nine patients had homozygous c.123_124insA mutation, and 1 patient had c.123_124insA and another nonsense mutation (547C>T). Umbilical cord separation occurred on the 14th day after birth or thereafter. Two patients had no severe infections owing to the prophylactic antibiotic treatment. Severe invasive bacterial infections occurred before the age of 3 in the other 8 patients. Among them, 7 patients had pneumococcal meningitis. Five patients died of invasive bacterial infection during infancy, although intravenous antibiotic treatment was started within 24 hours after onset in 4 patients among them. Analysis of cerebrospinal fluid of the patients who had fatal meningitis revealed very low glucose levels with only mild pleocytosis.

The clinical courses of invasive bacterial infections were often rapidly progressive despite the early, appropriate antibiotic treatment in IRAK4 deficiency patients. The early diagnosis and appropriate prophylaxis of invasive bacterial infections are necessary for the patients.

## INTRODUCTION

Invasive bacterial infections are still a major concern in clinicians. It is well known that a part of the patients with bacterial meningitis and sepsis patients develop rapidly progressive courses.

Interleukin (IL)-1 receptor-associated kinase 4 (IRAK4) plays an important role in the intracellular signal transduction from IL-1, IL-18, and Toll-like receptors (TLRs) other than TLR3.^1^ IRAK4 deficiency is an autosomal recessive primary immunodeficiency of the innate immune system.^2^ IRAK4-deficient patients suffer from severe invasive bacterial infections in early childhood.^3,4^*Streptococcus pneumoniae*, *Staphylococcus aureus*, and *Pseudomonas aeruginosa* are, by far, the most commonly isolated pathogens. A significant part of IRAK4-deficient patients have lethal pneumococcal meningitis. On the contrary, the occurrence of severe bacterial infections becomes less frequent with age.

We previously reported 2 siblings with IRAK4 deficiency, who had delayed separation of the umbilical cord.^5^ Although it is known that IRAK4-deficient patient often develop invasive bacterial infections, the clinical courses of the patients have not been well described. In this study, we applied a screening test for monocytic intracellular TNF-α production using a flow cytometer^5^ in patients who had severe bacterial infections, and analyzed the clinical characteristics of ten IRAK4-deficient patients.

## MATERIALS AND METHODS

### Flow Cytometric Screening Test

Peripheral blood cells were stimulated with lipopolysaccharide (LPS) (1 μg/mL) in the presence of brefeldin A (10 μg/mL) for 4 hours. Monocytic intracellular TNF-α production was analyzed by use of flow cytometry, as we described previously.^5^ The analysis gate was set for monocytes by side scatter and CD14 expression. This screening test was applied to the patients who had severe or recurrent invasive bacterial infections and their family members.

### Genetic Analysis of IRAK4 Gene

Each *IRAK4* exon was amplified by polymerase chain reaction, and sequenced with ABI PRISM 3100 Genetic Analyzer (Perkin-Elmer, Foster City, CA), as described elsewhere.^5^

This study was approved by the Institutional Review Board of Kyushu University Hospital. Informed consent was obtained from all participants.

## RESULTS

We identified 10 Japanese IRAK4-deficient patients from 6 families (Table 1). We reported patients 1 and 2 of family 1, previously.^5^ Five patients were diagnosed after death by *IRAK4* gene (accession number: NG_009892) sequencing. Nine patients had a homozygous c.123_124insA mutation which seemed to be a common *IRAK4* mutation in Japanese,^4,5^ and 1 patient (patient 4) had a c.123_124insA mutation and another nonsense mutation (547C>T) (data not shown). The time of umbilical cord separation was recorded in 8 patients (patients 1–8). Cord separation occurred on 14 days after birth or thereafter in these patients. Delayed separation of the umbilical cord (later than 21 days) was observed in 4 patients (50%). Five patients died of invasive bacterial infections in infancy. IRAK4-deficient patients in this study had no apparent abnormalities in serum immunoglobulin levels, lymphocyte subsets, NK activity, and lymphocyte proliferative response against phytohemagglutinin.

**TABLE 1:**
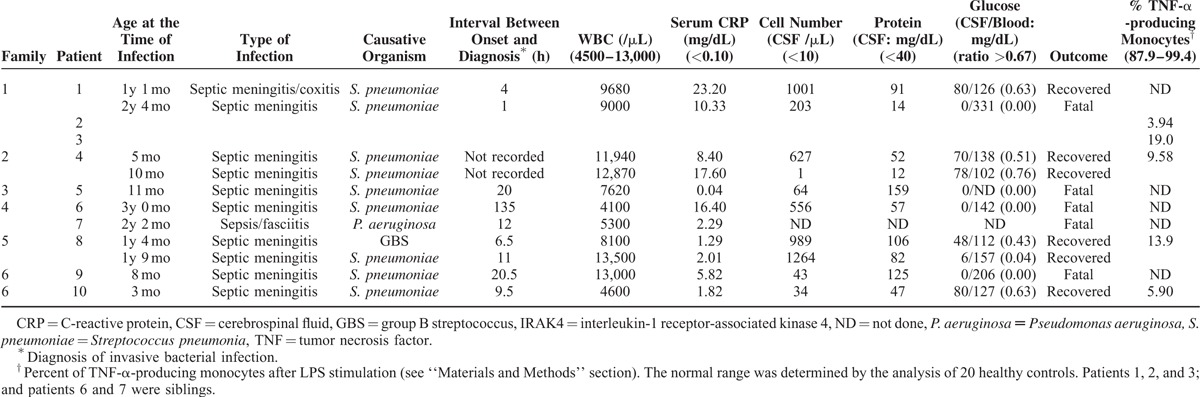
Demographic Data, Clinical Characteristics and Laboratory Findings of IRAK4-deficient Patients

Patients 2 and 3 have not suffered from invasive bacterial infections because of the antimicrobial prophylaxis since early infancy owing to the early diagnosis by the family history.^6^ Severe invasive bacterial infections occurred before the age of 4 years in the other 8 patients. Among them, 7 patients had pneumococcal meningitis. The diagnosis of invasive bacterial infection was made within 24 hours after onset in 8 episodes of infection, which led to the early intravenous antibiotic treatment. Five patients died of invasive bacterial infection, although intravenous antibiotic treatment was started within 24 hours after onset in 4 patients among them. Patients 5, 6, 7, and 9 died of first attack of invasive bacterial infections without prior episodes of infection.

Patients 1, 4, and 8 had meningitis 2 times. Meningitis was caused most commonly by *S. pneumoniae*. In patient 1, the second meningitis was fatal. On the contrary, patients 4 and 9 overcame meningitis twice without sequelae. Interestingly, the increase of the peripheral blood leukocyte number was not apparent in these IRAK4-deficient patients within 24 hours after onset (Table 1). Also, serum C-reactive protein (CRP) concentration was less than 5 mg/dL at the time of diagnosis in 5 out of 8 episodes of invasive bacterial infections within 24 hours after onset. Patients with marked pleocytosis recovered from bacterial meningitis (the first meningitis in patient 1, and the first and second meningitis in patient 8). Cerebrospinal fluid (CSF) glucose levels of the patients who had fatal course of bacterial meningitis were significantly lower than those of the patients who recovered (*P* < 0.05; Wilcoxon test and Mann–Whitney *U* test).

## DISCUSSION

In IRAK4 deficiency, the type of *IRAK4* mutation would not affect the characteristics of clinical manifestation significantly, because IRAK4-deficient patients have complete defect of IRAK4 protein in most cases.^1,4^ Although the c.123_124insA mutation has not been reported in other countries, it was observed in 19 of 20 alleles in IRAK4-deficient patients from 6 nonconsanguineous families in this study, suggesting the founder effect as the cause of the high frequency of this mutation in Japanese individuals. On the contrary, the c.123_124insA mutation was not found in 1000 Japanese individuals when we examined 1000 Genomes Project database of Kyoto University and in 400 Japanese individuals in the inhouse database of Kazusa DNA Research Institute, suggesting low prevalence of IRAK4 deficiency.

We previously reported that delayed separation of the umbilical cord is one of the clinical characteristics of IRAK4 deficiency.^4,5^ The date of umbilical cord separation is very often recorded in the maternal and child health handbook in Japan. Umbilical cord separation occurs within 7 days in most healthy neonates. Picard et al^4^ reported that separation of the umbilical cord later than 28 days after birth was observed in 10 among 48 patients with IRAK4 deficiency, despite race, which seemed to be consistent with the result of this study. The cause of delayed separation of the umbilical cord in IRAK4 deficiency is not known. It was reported that cord separation was accompanied with the infiltration of neutrophils progressively from the edges of the umbilical area towards the center in the first week of life in healthy children.^7^ No cells other than neutrophils were present and no bacteria were seen.^7^ Delayed separation of the umbilical cord in IRAK4-deficient patients might be associated with a low responsiveness of neutrophils to the unknown intrinsic ligand to cause neutrophil infiltration into the cord.

Pneumococcal meningitis was reported to be one of the most serious manifestations of IRAK4-deficient patients.^4^ It was the predominant cause of death in this study. Most patients were diagnosed as having invasive bacterial infections very early after the onset of symptoms, and the appropriate intravenous antibiotic treatments were conducted. Nonetheless, the course of invasive bacterial infections was fulminating in most cases. Unfortunately, the early and proper use of intravenous antibiotics, and intravenous gammaglobulin seemed to be almost totally ineffective in some cases (patients 7 and 9, and second invasive infection in patients 1 and 8). On the contrary, 4 patients (patients 1, 4, 8, and 10) showed complete recovery from bacterial meningitis at least once. The factor which contributed to the difference in the outcome of invasive bacterial infections in IRAK4-deficient patients has not been identified. Only patient 10 received pneumococcal vaccine before the invasive bacterial infection. Although he received the 13 valent conjugate vaccine only once, it might play an important role in host defense against fatal pneumococcal infection. In this study, we found that the patients who had a fatal course of pneumococcal meningitis showed low CSF glucose concentrations. The decreased CSF glucose concentration did not seem to be associated with the levels of pleocytosis at least in a part of IRAK4-deficient patients (Table 1, data not shown). It is possible that the lack of a strong inflammatory response in peripheral blood and CSF was due to the evaluation within early periods after the onset. However, the IRAK4-deficient patients might carry a high bacterial burden even at the onset of invasive bacterial infection due to the low host innate immune response, which might cause severe manifestations rapidly thereafter.

It was reported that severe invasive bacterial infections occur less frequently with age in IRAK4 deficiency. Prophylaxis of severe infections by antibiotics and vaccination has been reported to be very effective in all patients.^4^ The early diagnosis is pivotal to save IRAK4-deficient patients. By using our screening method for IRAK4 deficiency, we found that TNF-α producing monocytes after in vitro LPS stimulation^5^ were significantly decreased in all alive IRAK4-deficient patients (Table 1). Delayed separation of the umbilical cord may be the only sign for the early diagnosis of IRAK4 deficiency before the patients have highly invasive bacterial infections, in cases where they have no family history.
